# Effect of stoned olive pomace on rumen microbial communities and polyunsaturated fatty acid biohydrogenation: an *in vitro* study

**DOI:** 10.1186/s12917-014-0271-y

**Published:** 2014-11-26

**Authors:** Grazia Pallara, Arianna Buccioni, Roberta Pastorelli, Sara Minieri, Marcello Mele, Stefano Rapaccini, Anna Messini, Mariano Pauselli, Maurizio Servili, Luciana Giovannetti, Carlo Viti

**Affiliations:** Dipartimento di Scienze delle Produzioni Agro-alimentari e dell’Ambiente, Università di Firenze, Piazzale delle Cascine 18, 50144 Firenze, Italy; Centro di Ricerca per l’Agrobiologia e la Pedologia, Consiglio per la Ricerca e la Sperimentazione in Agricoltura, Piazza Massimo D’Azeglio 30, 50121 Firenze, Italy; Dipartimento di Scienze Agrarie, Alimentari e Agro-ambientali, Università di Pisa, Via del Borghetto 80, 56124 Pisa, Italy; Dipartimento di Biologia Applicata, Università di Perugia, Borgo XX Giugno 74, 06121 Perugia, Italy; Dipartimento di Scienze Economico-Estimative e degli Alimenti, Università di Perugia, Borgo XX Giugno 74, 06121 Perugia, Italy

**Keywords:** Stoned olive oil pomace, Sheep rumen microbiota, Fatty acid biohydrogenation, PCR-DGGE

## Abstract

**Background:**

Stoned olive pomace (SOP), which represents approximately 50% of the conversion process of olives to olive oil, is largely not utilised and creates costs for its disposal and has negative environmental impacts. *In vitro* trial experiments were employed to study the effect of feeds integrated with this bio-waste, which is rich in polyphenols, on rumen biohydrogenation, using sheep rumen liquor as inoculum.

**Results:**

Fatty acid (FA) analysis and a polymerase chain reaction denaturing gradient gel electrophoresis (PCR-DGGE) approach aimed at characterising the microbial community indicated that including SOP in feeds at the level of 50 g/kg and 90 g/kg induced changes in the FA profile and microbial populations. The simultaneous decrease of *Butyrivibrio proteoclasticus* and accumulation of vaccenic acid was observed. A depression in the populations of *Neisseria weaveri*, *Ruminobacter amylophilus* and other unclassified bacteria related to members of the Lachnospiraceae and Pasteurellaceae families was detected, suggesting that these microbial groups may be involved in rumen biohydrogenation.

**Conclusions:**

Supplementation of feeds with SOP alters the rumen bacterial community, including bacteria responsible for the hydrogenation of vaccenic acid to stearic acid, thereby modifying the FA profile of the rumen liquor. Hence, a use of SOP aimed to produce meat or dairy products enriched in functional lipids can be hypothesised.

## Background

The primary factor affecting ruminal biohydrogenation (BH) is the quality of the animal diet, the composition of which can affect the content of healthy fatty acids (FA) in milk and meat. In particular, the concentrate to forage ratio plays an important role in the accumulation of several BH intermediates, especially if the diet is rich in polyunsaturated fatty acids (PUFA) [1]. The inclusion of polyphenols in ruminant feeds has an inhibitory effect on the BH of dietary PUFA, as consequence of their influence on microbial activity and diversity [2]. This has been shown to increase the duodenal flow of bioactive FA, such as vaccenic acid (*trans*-11 C18:1, VA) and, as consequence, improve the nutritional value of milk fat from large and small dairy ruminants because this FA can be ∆^9^-desaturated to rumenic acid (*cis*-9,*cis*-12 C18:2, RA) in the mammary gland and other tissues. However, from the literature it is well known that the availability of VA in ruminant products is limited at the rumen level by its hydrogenation to stearic acid (C18:0, SA) or by the isomerisation to other C18:1 isomers by microbial activity taking place in the rumen [3].

The rumen microbial community is comprised of an enormous number of microbial species belonging to the Bacteria, Archaea and Eucarya domains. However, only a limited number of rumen microorganisms have been isolated and physiologically characterised thus far [4]. Among the different microbial species already identified, those belonging to the *Butyrivibrio* group appear of particular interest because they are known to be directly involved in BH [5]. Recent studies carried out on different species of ruminants have reported that diets enriched with polyphenols caused a decrease in SA and a simultaneous decline in key species of *Butyrivibrio* [2,6].

In the Mediterranean region, during the period of olive oil production, there is a high production of moist virgin olive pomace that presents a problem for its disposal. The most recent stoning virgin olive pomace techniques result in a residual product (stoned olive oil pomace, SOP) after the mechanical extraction of oil from olives followed by the drying of the cake on a fluid bed dryer. SOP is characterised by a high level of polyphenols (approximately 13 g/kg of dry matter) and low lignin content, which is considered to be the main factor that reduces the digestibility of olive pomace or olive cake when these by-products are utilised as animal feed [7-10]. These chemical characteristics render SOP potentially able to interfere with rumen fermentation [7]. However, little information is available in the literature on the effect of the SOP inclusion in ruminant diets on microbial strains involved in BH processes. The aim of the present study was to verify whether SOP supplementation in the sheep diet is able to affect the overall rumen microbial profile and, in particular, the *Butyrivibrio* group that influences the PUFA profile of rumen liquor (RL).

## Results

### Rumen liquor fatty acid composition

During the fermentation of the three feeds, the concentration of acetic acid (C2:0) did not exhibit significant differences with the exception of 12 h, when the percentage of this volatile fatty acid was higher in RL fermenting S5 and S9 (Table 1). Moreover, the presence of SOP in feeds significantly increased the concentration of propionic (C3:0), butyric (C4:0) and isovaleric (*iso* C5:0) acids compared to the content of these FA in RL with C. As consequence, the C2:0/C3:0 ratio in S5 and S9 was significantly lower than that in RL fermented with C at 12 and 24 h (Table 1).

**Table 1:** **Effect of stoned olive pomace (SOP) concentration (mM) on volatile fatty acid (VFA) production in ruminal fluid at 6, 12 and 24 h of incubation** ^**1**^

**VFA**	**Feed**		**Time (h)**		**SEM**	**P** ^**2**^
		**6**	**12**	**24**		**FXT**
C2:0	C	5.690 ^cβ^	7.360 ^bβ^	9.370 ^a^	0.240	0.021
	S5	6.700 ^cα^	8.020 ^bα^	9.190 ^a^		
	S9	3.690 ^cγ^	8.530 ^bα^	9.200 ^a^		
C3:0	C	2.450 ^β^	2.670 ^β^	3.010 ^β^	0.340	0.047
	S5	3.090 ^αβ^	3.250 ^αβ^	3.790 ^αβ^		
	S9	3.150 ^α^	3.990 ^α^	4.230 ^α^		
C4:0	C	3.050 ^cγ^	3.490 ^bγ^	3.840 ^aβ^	0.060	0.042
	S5	3.450 ^cβ^	3.750 ^bβ^	4.020 ^aα^		
	S9	3.670 ^cα^	3.980 ^bα^	4.150 ^aα^		
*iso* C4:0	C	0.156	0.189	0.193	0.075	0.083
	S5	0.135	0.147	0.153		
	S9	0.114	0.113	0.112		
C5:0	C	0.165	0.196	0.264	0.081	0.079
	S5	0.194	0.217	0.210		
	S9	0.105	0.233	0.235		
*iso* C5:0	C	0.350 ^bβ^	0.360 ^bγ^	0.500 ^aγ^	0.030	0.037
	S5	0.460 ^cα^	0.600 ^bβ^	0.680 ^aβ^		
	S9	0.490 ^bα^	0.800 ^aα^	0.820 ^aα^		
C2/C3	C	2.322 ^bα^	2.756 ^abα^	3.113 ^aα^	0.292	0.048
	S5	2.168 ^α^	2.680^αβ^	2.424 ^β^		
	S9	1.171 ^bβ^	2.138^aβ^	2.175 ^aβ^		

α, β, γ Within a column, means with different Greek superscripts are significantly different (P < 0.05); a, b, c within a row, means with different Latin superscripts are significantly different (P < 0.05). C = control feed; S5 = treatment with 50 g/kg of SOP; S9 = treatment with 90 g/kg of SOP.

^1^Mean values with their standard errors (SEM); number of samples for each treatment at any time = 3.

^2^Probability of significant effects due to the interaction of the experimental factors of Feed and Time (FXT).

C14:0 and C16:0 increased in rumen fluid incubated with S5, while with C and S9 the concentration of these FA significantly decreased (Table 2). S5 and S9 significantly increased the C13:0 concentration within 12 h, but at 24 h the percentage of this FA was significantly lower than that found in fermenters containing C (Table 2). C17:0 production was significantly depressed by S9, but not by S5, which was similar to C (Table 2).

**Table 2:** **Effect of the stoned olive pomace (SOP) concentration (g/100 g total fatty acids, FA) on medium chain fatty acid production in ruminal fluid at 6, 12 and 24 h of incubation** ^**1**^ **; number of samples for each treatment at any time = 3**

**FA**	**Feed**		**Time (h)**		**SEM**		**P** ^**2**^	**FA**	**Feed**		**Time (h)**		**SEM**		**P** ^**2**^
		**6**	**12**	**24**		**F**	**FXT**			**6**	**12**	**24**		**F**	**FXT**
C12:0	C	0.507 ^aαβ^	0.447 ^ab^	0.357 ^bαβ^	0.048	0.069	0.027	*anteiso* C15	C	0.510 ^β^	0.570 ^α^	0.523 ^β^	0.020	0.996	0.049
	S5	0.403 ^abβ^	0.363 ^b^	0.443 ^aα^					S5	0.477 ^bβ^	0.490 ^aβ^	0.626 ^aα^			
	S9	0.553 ^aα^	0.403 ^b^	0.320 ^cβ^					S9	0.700 ^aα^	0.463 ^bβ^	0.443 ^bγ^			
C13:0	C	0.973 ^bα^	1.133 ^bγ^	1.676 ^aα^	0.041	0.791	0.034	*iso* C16	C	0.143 ^α^	0.147^β^	0.123 ^β^	0.020	0.199	0.027
	S5	1.117 ^bα^	1.360 ^aβ^	1.340 ^aγ^					S5	0.106 ^bβ^	0.147 ^abβ^	0.173 ^aα^			
	S9	0.640 ^bβ^	1.526 ^aα^	1.463 ^aβ^					S9	0.146 ^αβ^	0.169 ^bα^	0.186 ^aα^			
C14:0	C	0.847 ^aβ^	0.823 ^aα^	0.753 ^bβ^	0.030	0.874	0.012	*iso* C17	C	0.110	0.136 ^β^	0.133 ^β^	0.030	0.002	0.044
	S5	0.663 ^bγ^	0.730 ^bβ^	0.913 ^aα^					S5	0.123 ^b^	0.183 ^aα^	0.176 ^aαβ^			
	S9	0.930 ^aα^	0.796 ^bαβ^	0.593 ^cγ^					S9	0.107 ^b^	0.183 ^aα^	0.193 ^aα^			
C16:0	C	5.440 ^aα^	5.063 ^bα^	4.957 ^bβ^	0.058	0.059	0.039	*anteiso* C17	C	0.143 ^bβ^	0.173 ^a^	0.174 ^aα^	0.007	0.041	0.042
	S5	4.570 ^cβ^	4.740 ^bβ^	5.537 ^aα^					S5	0.116 ^bγ^	0.133 ^b^	0.201 ^aα^			
	S9	5.780 ^aα^	4.327 ^bγ^	3.860 ^cγ^					S9	0.177 ^aα^	0.133 ^b^	0.101 ^cβ^			
C17:0	C	0.080 ^bβ^	0.093 ^b^	0.127 ^aα^	0.020	0.943	0.048	*cis*-9 C12:1	C	0.040 ^aα^	0.047 ^aα^	0.013 ^bγ^	0.030	0.061	0.002
	S5	0.073 ^bβ^	0.103 ^a^	0.120 ^aα^					S5	0.017 ^cβ^	0.036 ^bα^	0.050 ^aα^			
	S9	0.113 ^aα^	0.089 ^b^	0.088 ^bβ^					S9	0.037 ^aα^	0.020 ^bβ^	0.030 ^abβ^			
*iso* C13	C	0.081	0.111 ^β^	0.103 ^β^	0.020	0.610	0.048	*cis*-9 C14:1	C	0.277 ^bβ^	0.353 ^aα^	0.287 ^bαβ^	0.016	0.077	0.047
	S5	0.086	0.081 ^β^	0.087 ^β^					S5	0.233 ^bγ^	0.260 ^bβ^	0.363 ^aα^			
	S9	0.091^b^	0.173 ^aα^	0.167 ^aα^					S9	0.333 ^aα^	0.273 ^abβ^	0.250 ^bβ^			
*iso* C14	C	0.087 ^β^	0.087	0.097 ^β^	0.010	0.497	0.015	*trans*-9 C15:1	C	0.077 ^αβ^	0.076	0.053 ^β^	0.030	0.046	0.046
	S5	0.077 ^bβ^	0.091 ^b^	0.130 ^aα^					S5	0.053 ^bβ^	0.056 ^b^	0.093 ^aα^			
	S9	0.110 ^aα^	0.081 ^b^	0.081 ^bβ^					S9	0.100 ^aα^	0.057 ^b^	0.093 ^aα^			
*iso* C15	C	0.076 ^aα^	0.073 ^aαβ^	0.037 ^bβ^	0.010	0.275	0.047	*cis*-9 C16:1	C	0.087 ^bγ^	0.116 ^aβ^	0.103 ^abβ^	0.016	0.051	0.049
	S5	0.053 ^bβ^	0.080 ^aα^	0.076 ^aα^					S5	0.167 ^aα^	0.149 ^abα^	0.133 ^bα^			
	S9	0.073 ^α^	0.057 ^β^	0.060 ^α^					S9	0.123 ^β^	0.100 ^β^	0.093 ^β^			

α, β, γ Within a column, means with different Greek superscripts are significantly different (P < 0.05); a,b,c within a row, means with different Latin superscripts are significantly different (P < 0.05). C = control feed; S5 = treatment with 50 g/kg of SOP; S9 = treatment with 90 g/kg of SOP. ^1^Mean values with their standard error (SEM); ^2^Probability of significant effect due to the interaction of the experimental factors Feed and Time (FXT).

At the last point of sampling, the concentration of *iso* C15, *iso* C16 and *iso* C17 was significantly higher in S5 and S9 fermenters than in C (Table 2). With respect to C, *anteiso* C15 content was depressed during the fermentation of S9 and enhanced when S5 was fermented (Table 2). Moreover, the content of C17 *ante* increased during the fermentation of S5, whereas S9 exhibited the opposite trend (Table 2). The concentrations of *cis-*9 C12:1, *cis-*9 C14:1 and *trans-*9 C15:1 were characterised by an increasing trend in fermenters containing S5 (Table 2). When RL was incubated with S5, *cis*-11 C18:1 and *cis*-13 C18:1 increased significantly after 12 h compared to the fermenters containing C and S9 (Table 3). Moreover, S5 significantly decreased the BH rate of *cis*-9 C18:1, which exhibited the highest concentration at 24 h (Table 3). VA progressively accumulated during the entire fermentation period when SOP was added to feeds regardless of the percentage of inclusion, as consequence of a decrease in the extent of BH (Table 3). No significant differences among feeds were found for the other *trans* monoenes (Table 3). RA accumulated at 12 h in all cases but, when S5 and S9 were fermented, its percentage in RL was the highest according to a decrease in the BH rate (Table 3). In contrast, *trans*-10,*cis-*12 C18:2 was only detected at 12 h in S5 fermenters (Table 3). The BH rates of linoleic (*cis*-9,*cis*-12 C18:2, LA) and α-LNA (*cis*-9,*cis*-12,*cis*-15 C18:3) acids were similar in C and S9 (Table 3). It was simply lowered in S5, leading to a higher accumulation of LA and α-LNA at 24 h. Conjugated linolenic acid (*cis*-9,*trans*-11,*cis*-15 C18:3) and vaccelenic acid (*trans*-11,*cis*-15 C18:2) were detected at 24 h only in S9 fermenters (Table 3).

**Table 3:** **Effect of the stoned olive pomace (SOP) concentration on C18 fatty acid (g/100 g FA) production in ruminal fluid at 6, 12 and 24 h of incubation** ^**1**^ **; number of samples for each treatment at any time = 3**

**FA**	**Feed**		**Time (h)**		**SEM**	**P** ^**2**^	**FA**	**Feed**		**Time (h)**		**SEM**	**P** ^**2**^
		**6**	**12**	**24**		**FXT**			**6**	**12**	**24**		**FXT**
C18:0	C	2.377 ^cα^	2.590 ^bγ^	4.167 ^aα^	0.047	0.006	*trans*-11 C18:1	C	0.580 ^a^	0.350 ^bβ^	0.120 ^cβ^	0.021	0.005
	S5	2.203 ^cβ^	3.080 ^bα^	3.446 ^aβ^				S5	0.483 ^c^	0.677 ^bα^	0.850 ^aα^		
	S9	1.760 ^cγ^	2.806 ^bβ^	2.999 ^aγ^				S9	0.473 ^c^	0.653 ^bα^	0.830 ^aα^		
*cis*-9 C18:1	C	2.063 ^aβ^	1.610 ^bβ^	1.290 ^cβ^	0.023	0.032	*trans*-12 C18:1	C	0.047	0.047	0.053	0.024	0.485
	S5	1.950 ^aγ^	1.526 ^bγ^	1.567 ^bα^				S5	0.056	0.050	0.043		
	S9	2.563 ^aα^	1.663 ^bα^	1.163 ^cγ^				S9	0.053	0.036	0.029		
*cis-*11 C18:1	C	0.437	0.420	0.473	0.068	0.046	*cis*-9,*cis*-12 C18:2	C	4.527 ^aβ^	3.180 ^bα^	1.733 ^cβ^	0.043	0.031
	S5	0.340 ^b^	0.360 ^b^	0.527 ^a^				S5	3.750 ^aγ^	2.653 ^bβ^	2.060 ^cα^		
	S9	0.487	0.350	0.363				S9	4.780 ^aα^	2.523 ^bγ^	1.680 ^cβ^		
*cis*-12 C18:1	C	0.033 ^c^	0.150 ^aα^	0.110 ^bα^	0.010	0.006	*cis*-9,*trans*-11 C18:2	C	0.000 ^b^	0.021 ^aβ^	0.000 ^b^	0.020	0.033
	S5	0.050 ^b^	0.060 ^bβ^	0.117 ^aα^				S5	0.000 ^b^	0.112 ^aα^	0.000 ^b^		
	S9	0.040 ^b^	0.040 ^bβ^	0.073 ^aβ^				S9	0.000 ^b^	0.113 ^aα^	0.000 ^b^		
*cis*-13 C18:1	C	0.040 ^bβ^	0.070 ^aα^	0.050 ^bβ^	0.013	0.046	*trans*-10,*cis*-12 C18:2	C	0.000	0.000 ^β^	0.000	0.001	0.001
	S5	0.036 ^bβ^	0.040 ^bβ^	0.073 ^aα^				S5	0.000 ^b^	0.067 ^aα^	0.000 ^b^		
	S9	0.080 ^aα^	0.036 ^bβ^	0.053 ^bβ^				S9	0.000	0.000 ^β^	0.000		
*cis-*15 C18:1	C	0.020 ^β^	0.020	0.037	0.009	0.045	*cis*-9,*cis*-12,*cis*-15 C18:3	C	0.530 ^aβ^	0.393 ^bα^	0.283 ^cβ^	0.006	0.044
	S5	0.036 ^β^	0.036	0.033				S5	0.400 ^aγ^	0.357 ^bβ^	0.370 ^bα^		
	S9	0.053 ^aα^	0.023 ^b^	0.020 ^b^				S9	0.597 ^aα^	0.337 ^bγ^	0.283 ^cβ^		
*trans*-9 C18:1	C	0.037	0.040	0.040	0.008	0.045	*cis*-9,*trans*-11,*cis*-15 C18:3	C	0.000	0.000	0.000 ^β^	0.001	0.002
	S5	0.037	0.047	0.030				S5	0.000	0.000	0.000 ^β^		
	S9	0.037	0.050	0.027				S9	0.000 ^b^	0.000 ^b^	0.056 ^aα^		
*trans*-10 C18:1	C	0.047	0.040	0.053	0.011	0.910	*trans*-11,*cis*-15 C18:2	C	0.000	0.000	0.000 ^β^	0.001	0.004
	S5	0.060	0.063	0.057				S5	0.000	0.000	0.000 ^β^		
	S9	0.043	0.050	0.047				S9	0.000 ^b^	0.000 ^b^	0.143 ^aα^		

α, β, γ Within a column, means with different Greek superscripts are significantly different (P < 0.05); a, b, c within a row, means with different Latin superscripts are significantly different (P < 0.05). C = control feed; S5 = treatment with 50 g/kg of SOP; S9 = treatment with 90 g/kg of SOP. ^1^Mean values with their standard error (SEM); ^2^Probability of significant effect due to the interaction of the experimental factors Feed and Time (FXT).

### Microbial population profiling

**Figure 1:**
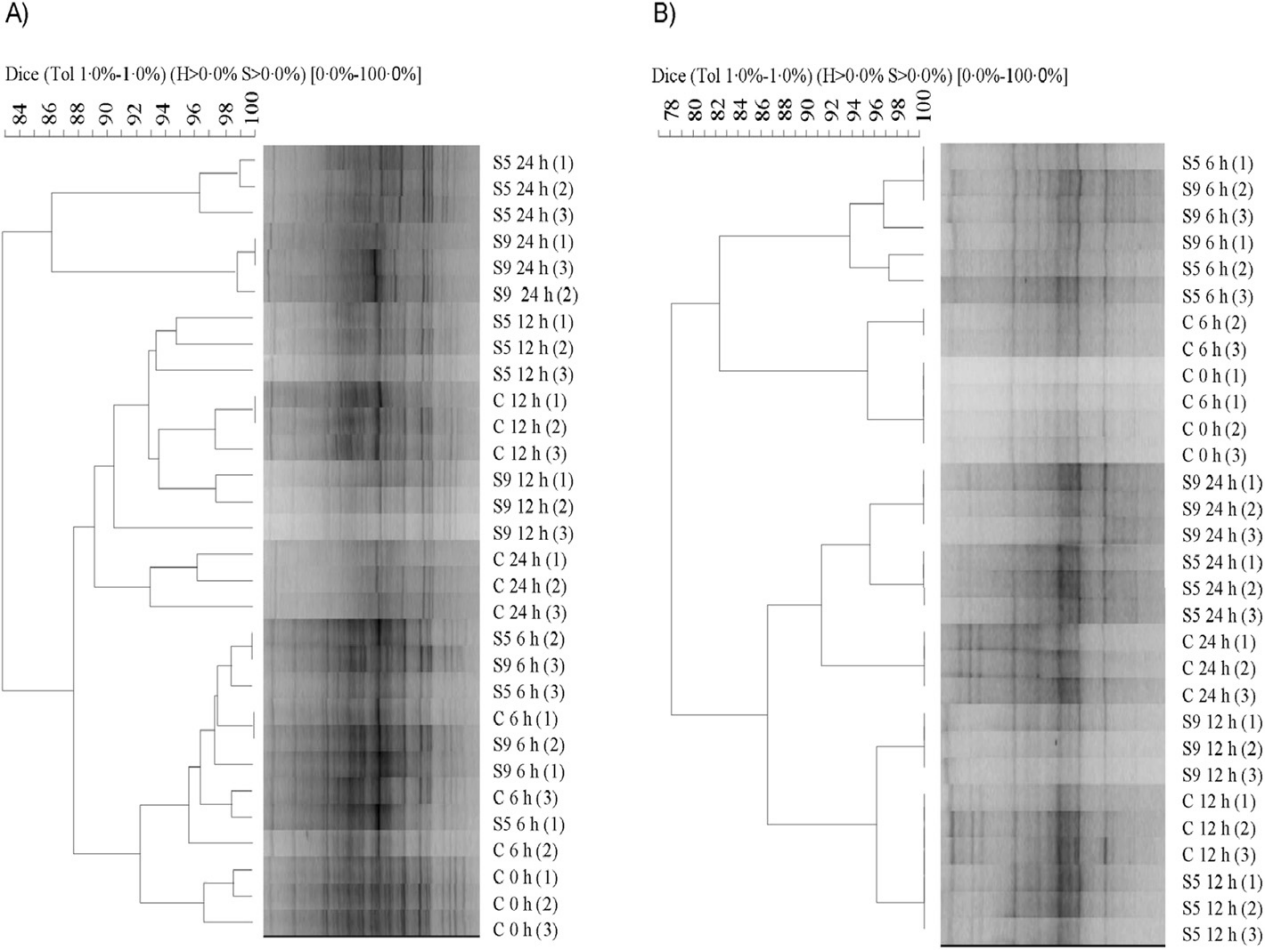
**Cluster analysis based on the unweighted pair group method with the arithmetic means of polymerase chain reaction denaturing gradient gel electrophoresis profiles demonstrating the effect of C, S5 and S9 diets on the total bacteria (A) and** ***Butyrivibrio*** **group (B) in rumen liquor collected at 0, 6, 12 and 24 h.** C = control feed; S5 = treatment with 50 g/kg of stoned olive pomace; S9 = treatment with 90 g/kg of stoned olive pomace. Scale relates to percent similarity.

DGGE analysis of PCR-amplified partial 16S rRNA genes was performed on the total bacteria, and *Butyrivibrio* populations of RL incubated with the three diets. Microbial profiles obtained using universal primers for bacteria displayed a complex band pattern in all samples (Figure 1A). A UPGMA dendrogram separated samples incubated with S5 and S9 diets and collected at 24 h from all the other samples, with 82.8% similarity (Figure 1A). Within the cluster containing S5 and S9 and collected at 24 h two subclusters (86.2% similarity) were evident, based on the percentage of SOP (Figure 1A). Samples collected at 0 and 6 h formed a different group when compared with samples collected at 12 h and with control samples collected at 24 h, with a similarity of 87.6%. A similarity higher than 92% was found in RL samples inoculated with C, S5 and S9 collected at 0 and 6 h (Figure 1A). According to the AMOVA of DGGE banding patterns, there was a significant effect of sampling time on bacterial communities (percentage of variation = 31.29; P < 0.01) and a significant effect of the diet within samples collected at the same time point (percentage of variation = 22.29; P < 0.001).

PCR-DGGE analysis of members of the *Butyrivibrio* group exhibited a less complex pattern than the total bacteria (Figure 1B). Two main clusters were evident, separating all samples collected at 0 and 6 h from those collected at 12 and 24 h, with 77.0% similarity (Figure 1B). Subclusters once again clearly reflected the percentage of the amount of SOP added and the collection time (Figure 1B). Control samples collected at 0 and 6 h grouped differently from samples incubated with S5 and S9 diets (Figure 1B), with 81.2% similarity. Moreover, all samples collected at 12 h grouped separately from those collected at 24 h, with 85.6% similarity (Figure 1B). Along within the latter group, samples to which the S5 and S9 diets were added grouped together, separately from control samples, with 90.6% similarity (Figure 1B). AMOVA analysis indicated a significant effect of sampling time on *Butyrivibrio* DGGE banding patterns (percentage of variation = 58.67; P < 0.01) and of the diet within samples collected at the same time point (percentage of variation = 24.49; P < 0.001).

### Sequence analysis of bacterial and *Butyrivibrio*-specific PCR-DGGE bands

**Figure 2:**
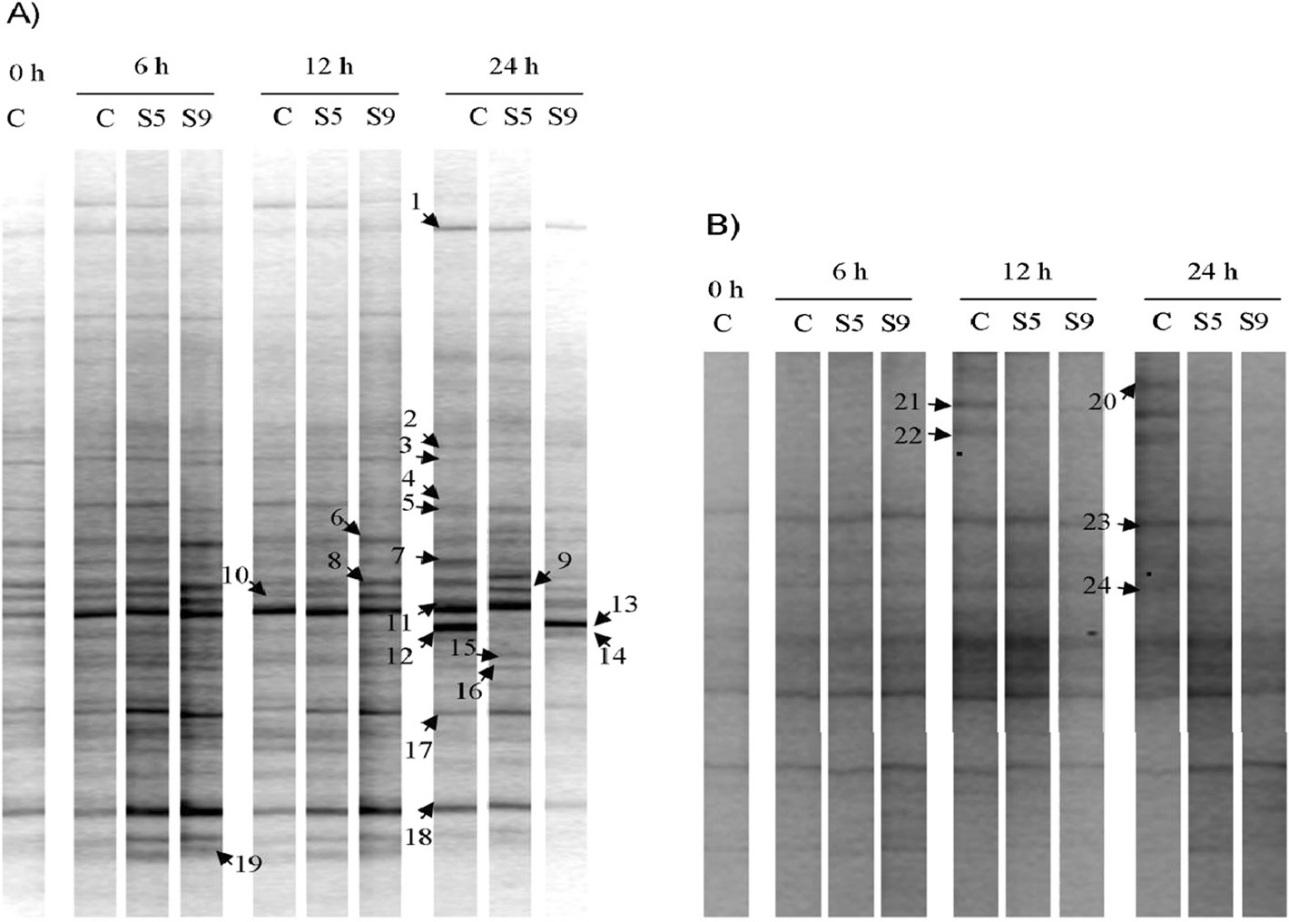
**PCR-DGGE profiles of the total bacterial community (A) and** ***Butyrivibrio*** **members (B) in rumen liquor inoculated with the C, S5 and S9 diets and collected at 0, 6, 12 and 24 h.** C = control feed; S5 = treatment with 50 g/kg of stoned olive pomace; S9 = treatment with 90 g/kg of stoned olive pomace. Bands indicated by numbers were selected for sequencing.

PCR-DGGE bands exhibiting remarkable changes in response to SOP in total bacterial or *Butyrivibrio* populations (bands 1, 5, 7, 8, 9, 11, 12, 17, 18, 20, 21, 22, 23 and 24) were excised, re-amplified and sequenced (Figure 2). Moreover, to gain more information on the composition of the rumen bacterial community of sheep, ten bands obtained with primers F968/R1401 for total bacteria (bands 2, 3, 4, 6, 10, 13, 14, 15, 16 and 19) were selected and sequenced, even if their intensity was not affected by SOP (Figure 2A). Putative taxonomic identification for each band subjected to sequencing is reported in Table 4.

**Table 4:** **Identification of selected polymerase chain reaction denaturing gradient gel electrophoresis (PCR-DGGE) fragments**

**PCR-DGGE band**	**Nearest match (GenBank accession no.; % sequence similarity)**	**Taxonomic classification**
Total bacterial community analysis		
1	*Pasteurella testudinis* (NR_042889; 90%)	Unclassified Pasteurellaceae
2	*Bergeriella denitrificans* (NR_040933; 99%)	*Bergeriella denitrificans*
3	*Bergeriella denitrificans* (NR_040933; 99%)	*Bergeriella denitrificans*
4	*Clostridium lavalense* (NR_044289; 93%)	Unclassified Clostridiaceae
5	*Neisseria weaveri* (NR_025902; 99%)	*Neisseria weaveri*
6	*Neisseria weaveri* (NR_025902; 98%)	*Neisseria weaveri*
7	*Neisseria weaveri (*NR_025902; 99%)	*Neisseria weaveri*
8	*Neisseria weaveri* (NR_025902; 98%)	*Neisseria weaveri*
9	*Neisseria flavescens* (KF030235; 100%)	*Neisseria flavescens*
10	*Clostridium citroniae* (NR_043681; 90%)	Unclassified Clostridiaceae
11	*Ruminobacter amylophilus* (NR_026450; 99%)	*Ruminobacter amylophilus*
12	*Neisseria flavescens* (KF030235; 100%)	*Neisseria flavescens*
13	*Neisseria flavescens* (KF030235; 100%)	*Neisseria flavescens*
14	*Neisseria weaveri* (NR_025902; 98%)	*Neisseria weaveri*
15	*Howardella ureilytica* (NR_044022; 94%)	Unclassified Clostridiaceae
16	*Roseburia faecis* (NR_042832; 90%)	Unclassified Lachnospiraceae
17	*Butyrivibrio hungatei* (NR_025525; 90%)	Unclassified Lachnospiraceae
18	*Butyrivibrio hungatei* (NR_025525; 93%)	Unclassified Lachnospiraceae
19	*Ruminococcus torques* (NR_036777; 90%)	Unclassified Lachnospiraceae
*Butyrivibrio*-specific analysis		
20	*Butyrivibrio proteoclasticus* (NR_102893; 92%)	Unclassified Lachnospiraceae
21	*Butyrivibrio proteoclasticus* (NR_102893; 98%)	*Butyrivibrio proteoclasticus*
22	*Butyrivibrio proteoclasticus* (NR_102893; 99%)	*Butyrivibrio proteoclasticus*
23	*Robinsoniella peoriensis* (NR_041882; 94%)	Unclassified Lachnospiraceae
24	*Eubacterium ruminantium* (NR_024661; 92%)	Unclassified Lachnospiraceae

**Table 5:** **Composition of feeds used as substrates for the fermentation and the main fatty acids (FA) in rumen liquor (RL) at the start of fermentation**

**Feed composition**	**C**	**S5**	**S9**
**Ingredients (g/kg DM)**			
Grass hay	103.45	103.45	98.04
Wheat straw	103.45	103.45	98.04
Mais meal	545.52	510.00	504.80
Soybean meal	42.76	42.76	40.52
Wheat bran	33.10	33.10	31.37
Bean flakes	20.69	20.69	19.61
Soybean flakes	12.41	12.41	11.76
Horsebean flakes	11.03	11.03	10.46
Barley	109.66	95.17	78.43
Stoned olive oil cake	---	50.00	90.00
Maize germ meal	17.93	17.93	16.99
**Chemical composition**			
Dry Matter kg	724.70	719.00	713.0
**expressed as g/kg of DM**			
Crude protein (6.25 x N)	115.91	116.23	116.40
Crude fat	23.42	24.51	25.63
Neutral detergent fibre	366.00	379.40	391.81
Acid detergent fibre	194.73	205.63	215.82
Lignin	81.64	81.81	82.53
Ash	58.76	61.25	63.56
Non protein nitrogen	2.48	4.43	5.27
Soluble protein	8.57	9.72	10.39
Neutral detergent insoluble protein	2.50	5.60	7.99
Acid detergent insoluble protein	1.11	2.30	3.21
**Main fatty acids in RL at the start**			
**of fermentation (g/100 g of total FA)**			
C16:0	18.05	17.23	16.33
C18:0	1.90	1.79	1.79
*cis-9* C18:1	23.92	25.19	26.81
*cis*-9,*cis*-12 C18:2	52.96	52.98	52.38
*cis*-9,*cis*-12,*cis*-15 C18:3	2.67	2.44	2.32

C = control feed; S5 = treatment with 50 g/kg of stoned olive pomace (SOP); S9 = treatment with 90 g/kg of SOP.

**Figure 3:**
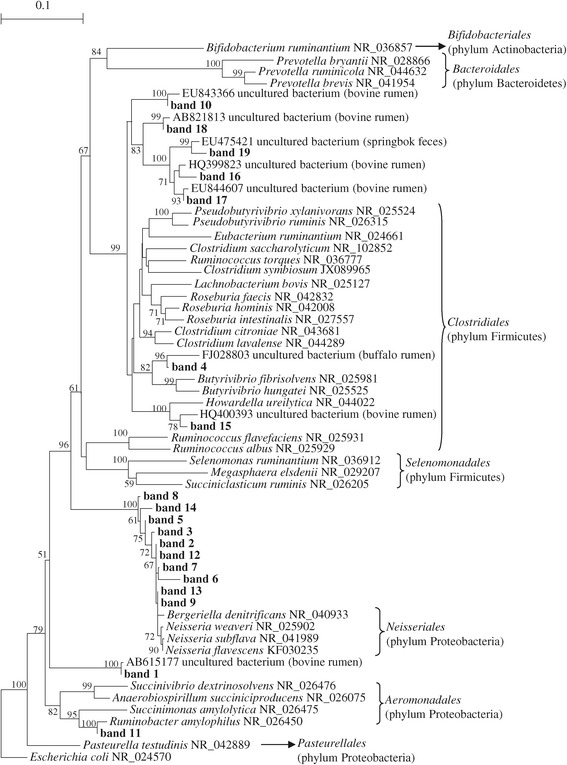
**Neighbour-joining tree built using all 16S rRNA sequences obtained from the total bacteria PCR-DGGE gels and sequences of rumen bacteria of equivalent length, retrieved from the GenBank database.** Sequences obtained in this study are indicated in boldface. Bootstrap values >50% based on 1000 replications are indicated at the nodes. The 16S rRNA gene sequence of *Escherichia coli* (NR_024570) was selected as the outgroup.

Phylogenetic analysis of the nineteen sequences of the PCR-DGGE fragments obtained with primers F968/R1401 (total bacteria) and sequences from rumen bacteria of equivalent length retrieved from the GenBank database was performed. The results indicated that seven sequences (bands 4, 10, 15, 16, 17, 18 and 19) were related to known species of *Clostridiales* (Figure 3), ten sequences (bands 2, 3, 5, 6, 7, 8, 9, 12, 13 and 14) were related to *Neisseriales* and the remaining two sequences were related to *Pasteurellales* (band 1) and *Aeromonadales* (band 11) (Figure 3, Table 4).

The analysis of total bacterial PCR-DGGE profiles evidenced that the intensities of seven bands, corresponding to *Neisseria weaveri* (bands 5, 7 and 8), *Ruminobacter amylophilus* (band 11), unclassified Pasteurellaceae (band 1) and Lachnospiraceae (bands 17 and 18) were reduced at 24 h in samples receiving the S9 diet in comparison to controls, whereas one band, identified as *Neisseria flavescens* (band 9), increased in S9 samples at the same sampling time (Figure 2A). On the contrary, minor differences were observed in presence of the S5 diet at 24 h in comparison to controls because only disappearance of band 12 (*Neisseria flavescens*) and the appearance of band 9 (*Neisseria flavescens*) were detected (Figure 2A).

**Figure 4:**
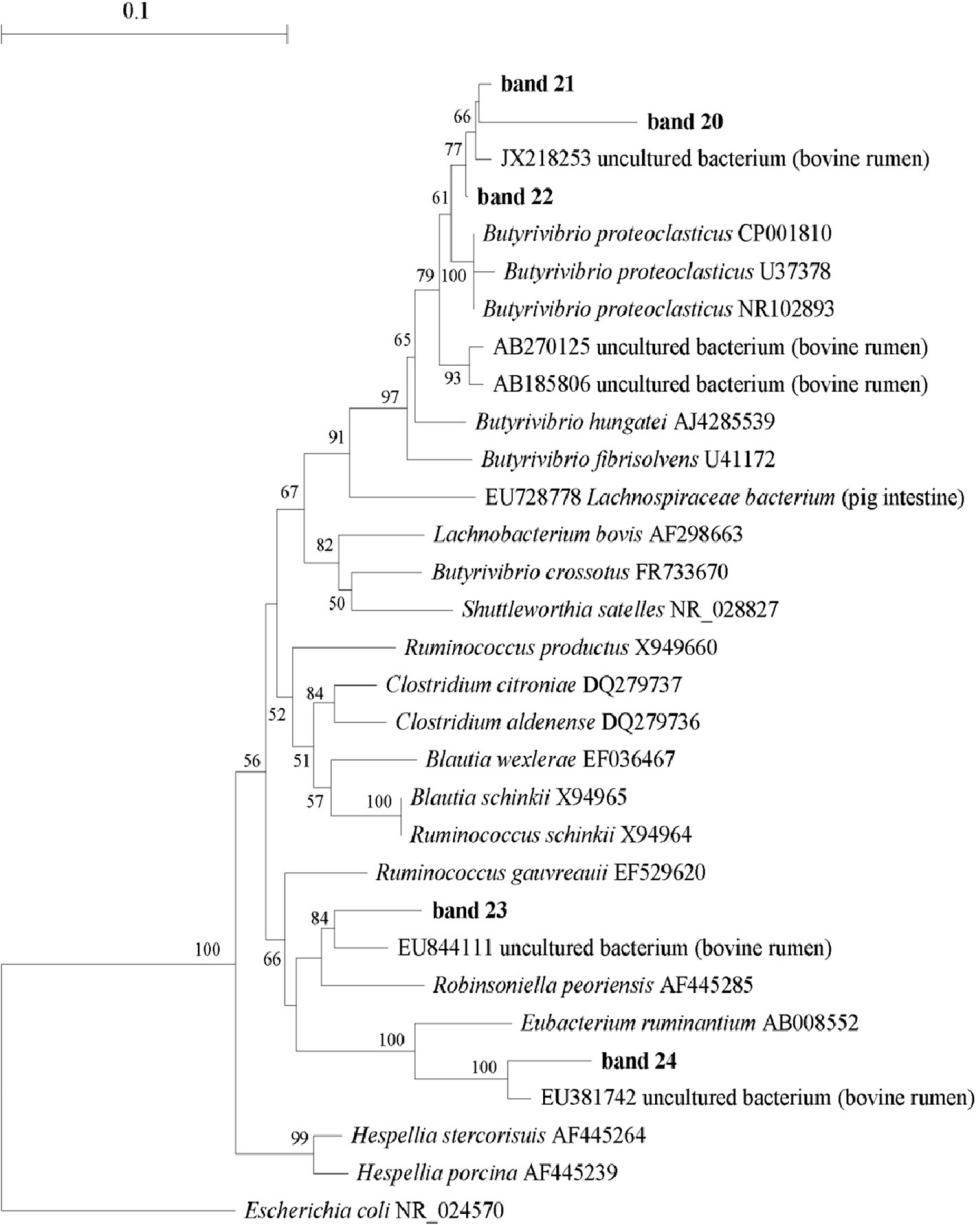
**Neighbour-joining tree built using all 16S rRNA sequences obtained from** ***Butyrivibrio*** **-specific PCR-DGGE gels and sequences of rumen bacteria of equivalent length, retrieved from the GenBank database.** Sequences obtained in this study are indicated in boldface. Bootstrap values of >50% based on 1000 replications are indicated at the nodes. The 16S rRNA gene sequence of *Escherichia coli* (NR_024570) was selected as the outgroup.

A phylogenetic tree was also constructed with the five sequences obtained with the *Butyrivibrio*-specific primers F968/Bfib and other sequences of equivalent length, representative of bacterial species related to the Lachnospiraceae family. As displayed in Figure 4, two sequences (bands 21 and 22) grouped with sequences representative of *Butyrivibrio proteoclasticus,* whereas three sequences (band 20, 23 and 24) displayed a very low level of similarity with other known bacterial species belonging to the Lachnospiraceae family.

PCR-DGGE profiles obtained using *Butyrivibrio*-specific primers exhibited weak changes in the *Butyrivibrio* community in relation to diets. In S5 and S9 samples, the intensities of bands 21 and 22, identified as *Butyrivibrio proteoclasticus*, were reduced at 12 h when compared to C (Figure 2B). Moreover, considering samples collected at 24 h, band 20, (unclassified Lachnospiraceae), exhibited a lower intensity than the controls after incubation with the S5 and S9 diets, whereas the intensities of bands 23 and 24 (unclassified Lachnospiraceae) decreased only slightly in the presence of S9 diet (Figure 2B).

## Discussion

In the literature it is well known that the inclusion of polyphenols in ruminant diets affects rumen metabolism, decreasing dietary protein degradation and fatty acid BH by means of targeting specific groups of microorganisms [2,6,10,11]. The *in vitro* degradability of organic matter, which is strongly related to microbial activity in the fore-stomach, is typically low for olive oil cakes on the basis of their polyphenol content. In fact, when dietary polyphenols are inactivated by using poly ethylene glycol (PEG) no detrimental effect has been observed in rumen microorganisms [10,12,13]. SOP contains a high level of polyphenols, and it is hypothesised that this supplement might be useful for improving the content of PUFA in products derived from ruminant livestock, at the same time contributing to the environmental sustainability of animal productions [7,10]. However, until now, there has been a lack of knowledge on the effect of SOP on lipid metabolism and rumen microbial communities involved in fatty acid BH processes. This information is essential to optimise the employment of SOP in ruminant feeding.

Our findings indicated that the inclusion of SOP in feeds stimulated the production of volatile fatty acids (VFA), suggesting that microbial activity was modified by the presence of SOP in feeds: the highest increase of C3:0 in the fermented RL inoculated with S5 and S9 can be related with a good level of amylolytic bacteria activity, while the constant production of C2:0 and the increase of *iso* C5, arising from microbial degradation of dietary amino acids, can be an indication of stimulated cellulolytic bacteria activity [10]. *Iso* C5 is the precursor of *iso* C15 and *iso* C17, which arise from rumen cellulolytic bacteria metabolism [14]. In our experiment, *iso* C15 and *iso* C17 production was stimulated by SOP, confirming that cellulolytic activity was not perturbed. The literature contains controversial results regarding the stimulating or depressing effects of olive oil by-products on rumen VFA production [12,15,16]. In their *in vitro* study Martin et al. [12] reported low levels of VFA when olive cake was fermented with rumen sheep fluid, while Yanetz-Ruitz et al. [16] obtained values higher than those reported in the former study. These differences are most likely strongly related to the olive variety, different oil extraction procedures used to obtain olive cakes and to the associative effects of the olive by-product with other dietary components.

SOP supplementation in feeds did not protect the double bond *cis*-9 from saturation, as demonstrated by the BH of OA and RA, which decreased during the fermentation period. A decrease in the isomerisation of OA to other *trans* C18:1 isomers [17] was hypothesised because no significant variations in the concentrations of these monoenes were detected. The temporary RA accumulation at 12 h in RL fermented with S5 and S9 can be related to a negative feedback effect caused by VA accumulation in these fermenters. This hypothesis can also be extended to conjugated linolenic acid and vaccelenic acid, further precursors of VA from α-LNA biohydrogenation, which appeared only at the end of fermentation in fermenters containing the highest content of SOP. VA accumulation in RL fermented with S5 and S9 is closely related to a decrease in *Butyrivibrio proteoclasticum* growth, as revealed by PCR-DGGE analysis. SOP did not contribute to the protection of LA and α-LNA from isomerisation to their *cis-*9,*trans-*11 isomers, indicating that LA-Isomerase activity is not influenced by SOP inclusion in feeds. Moreover, the shift of LA and α-LNA BH toward the *trans-*10 isomer was not enhanced. This trend agrees with several studies that demonstrated polyphenols do not favour the increase in the concentrations of *trans*-10 monoenes, whose synthesis is strongly related to the starch content of the diet [2,6,7].

In this study we employed a PCR-DGGE approach to evaluate the effect of SOP on rumen bacterial and *Butyrivibrio* communities. PCR-DGGE provides information on the predominant populations in the community and, using this technique it is possible to detect the sequences of the prevalent bacterial populations without the need for large clone libraries [18]. Nevertheless, PCR-DGGE presents some limitations: a single DGGE band may represent several species with identical partial DNA sequences [19], or several bands could be generated from a single organism because of multiple, heterogeneous operons [20]. However PCR-DGGE widely used in microbial ecology investigations.

Cluster analysis of PCR-DGGE profiles obtained with universal primers for the 16S rRNA gene clearly indicated a shift in the total bacterial community in the presence of SOP-enriched diets in comparison to controls, as confirmed by AMOVA. Buccioni et al. [2] demonstrated that polyphenols, such as tannins, affected the FA composition of the sheep rumen bacterial community, suggesting changes in its composition and/or activity in relation to the BH process. In our study, the effect of SOP on rumen bacterial communities appeared to depend on the level of its supplementation in the diet and the incubation time. Indeed, after 24 h of incubation with 90 g/kg of SOP, some bands in the PCR-DGGE profiles exhibited a decreased intensity. Thus the changes observed in the PCR-DGGE banding patterns may reflect the reduced abundance of the most sensitive species of ruminal bacteria to the antimicrobial action of SOP. Our observation is in agreement with previous *in vitro* studies, supporting the idea that polyphenols from different plants can reduce the activity and proliferation of different ruminal microorganisms [21]. The inhibitory effect exerted by these compounds has been explained by their ability to form complexes with the bacterial wall and to inactivate many extracellular enzymes [22].

Until now few studies have been carried out on sheep rumen microbiota using PCR-DGGE analysis followed by sequencing and identification of the dominant bacterial groups. In this study, only eleven PCR-DGGE bands obtained from the total bacterial community analysis were highly related to the 16S rRNA genes of known species, whereas the other bands corresponded to yet unclassified bacteria. This result is not surprising because the use of different culture-independent methods has demonstrated that the rumen microbiota is more diverse than previously hypothesised by considering the number of cultivated species [4]. On the whole, the sequenced bands were related mainly to species belonging to the Clostridiaceae family and the genus *Neisseria*. The first taxonomic group includes many cellulolytic and amylolytic species, which are often found in the rumen [4]. In contrast, only a Gram-negative carbohydrate-fermenting bacterium similar to *Neisseria* has been isolated from sheep rumen [23]. However, because members of the Neisseriaceae family are mammalian commensals [24], their presence in the rumen is plausible. In the analysed samples, we also detected the presence of *Ruminobacter amylophilus,* a typical rumen bacterium that may occur in reasonably large numbers in high grain or high roughage diets [25].

The most interesting changes in PCR-DGGE profiles were observed for the *Neisseria flavescens, Neisseria weaveri, Ruminobacter amylophilus* species and for members of the Lachnospiraceae and Pasteurellaceae families at 24 h in RL inoculated with S9 diet. Previous *in vitro* and *in vivo* studies have indicated that some members of the Lachnospiraceae family, such as *Butyrivibrio* species, are the main known bacteria involved in rumen BH [26]. Nevertheless, Huws et al. [27] have recently suggested that other yet not known bacterial species may play an important role in the BH process, via analysis of the RL of dairy cows using T-RFLP and DGGE approaches. Thus, the findings thus far from studies on pure cultures may not be sufficient to explain the bacterial contribution to rumen BH *in vivo,* which appears more complex than previously thought. For example, a previous study by Hudson et al. [28] has indicated that some bacterial species, such as *Staphylococcus* spp. and *Streptococcus bovis*, have the capacity to hydrate specific CLA intermediates, diverting them from the BH pathway. Our results strengthen the hypothesis of Huws et al. [27], despite the fact that additional investigations carried out with advanced techniques such as metagenomics and metatranscriptomics could better clarify the potential role of the different bacterial groups in the FA metabolism of sheep rumen.

Previous *in vitro* experiments have demonstrated that members of the *Butyrivibrio* group are able to biohydrogenate unsaturated FAs more rapidly than other species, and that only *B. proteoclasticus* has been recognised to reduce C18:1 to C18:0 [5]. Therefore we performed a *Butyrivibrio*-specific PCR-DGGE analysis to investigate the effect of SOP supplementation on this taxonomic group in detail, although the *Butyrivibrio* group comprises only a minor part of ruminal bacteria [29]. Cluster analysis indicated that both diets supplemented with SOP affected the composition of the *Butyrivibrio* population. Indeed, at both 12 and 24 h of incubation we observed a reduced intensity in specific PCR-DGGE bands. Sequence analysis revealed that two bacterial groups responding negatively to SOP after 12 h of incubation were closely related to *B. proteoclasticus* (levels of 16S rDNA similarity above 98.0%), which is the only cultivable SA producer. A significant increase in VA was only observed in relation to the incubation time in samples to which SOP had been added. Therefore, we hypothesise that SOP is able to decrease the hydrogenation of *trans* C18:1 and *trans* C18:2 intermediates by negatively affecting the growth of *B. proteoclasticus* or other species of *Butyrivibrio* not identified here. Our findings agree with the results obtained by Vasta et al. [6], who found a correlation between the reduced abundance of *B. proteoclasticus* and the simultaneous increase in VA in lamb rumen fluid, following the addition of quebracho tannins to the diet. However, the other few studies available in the literature on the effect of plant extracts rich in polyphenols on ruminal microorganisms have presented contrasting results. According to Ghaffari et al. [30], phenolic compounds from pistachio by-products used in the diet of sheep did not affect the abundance of *B. proteoclasticus* in rumen fluid. Moreover, extracts from 37 Australian plants containing polyphenols selectively inhibited *B. proteoclasticus,* and only some of them affected *B. fibrisolves* [31]. The variable effect of plant extracts on the members of *Butyrivibrio* group could be related to the type of polyphenols they contain and the supplementation level in the diet, as previously suggested [11].

## Conclusions

Supplementation of feeds with SOP inhibited the rumen BH of C18 unsaturated FAs in a dose dependent manner, resulting in a decrease in the SA concentration and in an increase in VA. In particular, changes in rumen fatty acid profiles were associated with changes in the bacterial community, including bacteria responsible for the hydrogenation of VA to SA.

## Methods

### Feed composition

Feeds used as the substrates of the fermentation were: a control diet (C) in which the SOP was not included and other two diets (S5 and S9) in which the integration with SOP was 50 g/kg of DM and 90 g/kg of DM, respectively. The amount of SOP used in this experiment was chosen with the criterion of practicality under farm conditions (the diets used in this trial were formulated on the basis of previous *in vivo* trials with Chianina bulls and dairy Comisana sheep; data unpublished). The diets were formulated to be isoproteic and isoenergetic. The ingredients and chemical compositions of the feeds are displayed in Table 5. SOP was obtained after mechanical extraction of virgin olive oil using the following operating conditions [7]: the olives were stoned and malaxed for 40 min at 25°C, and the oil extraction was performed using a three phase decanter (mod. 400 ECO, RCM Rapanelli Costruzioni Meccaniche s.r.l., Bevagna, PG, Italy). After storage at room temperature for 36 hours, stoned olive cake was dried using a fluid bed dryer; the initial temperature of the drying air flow was 120°C and the maximum temperature of olive cake during the drying process was 45°C. The dried stoned olive cake was stored at room temperature. The proximate composition (according to A.O.A.C procedures [32]) of SOP was: DM (873.80 g/kg), crude protein (118.31 g/kg of DM), neutral detergent fibre (490.51 g/kg of DM), acid detergent fibre (347.40 g/kg of DM), acid detergent lignin (85.61 g/kg of DM) and 63.43 g/kg of DM of crude fat in which the main FA contained were C16:0 (12.81 g/100 g of total FA), *cis-9* C18:1 (76.43 g/100 g of total FA) and *cis-9,cis-12* C18:2 (6.82 g/100 g of total FA). The polyphenol composition of SOP was determined according to Servili et al. [9]: 3,4-dihydroxyphenolethanol (1.16 g/kg DM), 4-hydroxyphenolethanol (0.11 g/kg DM); p-coumaric acid (0.04 g/kg DM), verbascoside (1.33 g/kg DM), 2-(3,4-hydroxyphenyl)ethyl(3S,4E)-4-formyl-3-(2-oxoethyl)hex-4-enoate (1.16 g/kg DM). The total polyphenol content in SOP was 3.80 g/kg DM.

### *In vitro* incubation with sheep ruminal fluid

The *in vitro* incubation was performed according to Tedeschi et al. [33] with several modifications. Four sheep, conditioned with a basal diet formulated to shape rumen microflora and composed of grass hay (770 g/kg DM), soybean meal (55 g/kg DM), barley meal (175 g/kg DM), were used to provide rumen contents. Animals had continuous access to water and mineral blocks. After a 4 week adaptation period, approximately 1 litre of rumen contents was collected from each sheep using a rumen fluid sampling pump on the same day before the morning meal. The handling of the animals was performed according to the Institutional Animal Care and Use Committee of Florence University (IACUC, 2004). The RL was immediately mixed with CO_2_ to avoid O_2_ contamination and transferred to the laboratory in a thermostatic box (39°C) under anaerobic conditions. The RL was then filtered through four layers of cheesecloth into a flask under a continuous flow of CO_2_. An aliquot of the RL was buffered (1:3, v/v) by adding an artificial saliva solution [34]. Feeds (2 g of DM) were incubated in triplicate with 200 ml of inoculum. The incubator consisted of a thermostatic chamber (39°C) equipped with twenty-seven 300 ml glass fermentation vessels provided with two inlets (one to release gas through a valve and one for the pH probe) and connected to an electronic pressure transducer (pre-set at 65 kPa) and an electronic gas valve. When the inside gas pressure reached the pre-set value, the valve was opened, releasing approximately 2 ml of gas. The fermentation pattern was monitored with PC software (Labview 5.0, National Instr., Austin, TX). Each vessel containing substrate inoculated with rumen fluid saturated with CO_72_ to guarantee anaerobic conditions was continuously stirred. Samples of RL were collected at 6, 12 and 24 h of incubation. At each sampling time, three vessels per treatment were used for the microbial characterisation and FA analysis as follows: the entire contents of each vessel were separated into four aliquots; three aliquots of 1 ml and one aliquot of 150 ml were stored at −80°C for DNA extraction and FA profile determination, respectively, as described below. Each vessel was considered to be a single experimental unit according to Buccioni et al. [2,35]

Samples of RL immediately after the addition of buffer solution (200 ml) and before feed inoculation (as blank to control the quality) and samples of RL (200 ml) inoculated with feeds, at the start of fermentation (t = 0 min), were collected in triplicate for FA profile analysis. The fat content of the RL blank was very low (0.01 g/l), as a consequence of the procedure adopted for the preparation of the inoculum; hence, the initial contribution of RL to FA composition of the inoculum was negligible (data not shown). Table 5 displays the FA composition of RL inoculated with the three diets at the beginning of fermentation. In the feeds the concentration of oleic acid (*cis-*9 C18:1, OA) increased according to the percentage of SOP inclusion in the diet.

### Feed proximate analysis

Samples of feeds were oven-dried at 60°C for 24 h. The dry samples were analysed for crude protein, ash and crude fat according to the 954.01, 954.05 and 920.39 procedures of AOAC (1990) [32], respectively. Neutral detergent fibre, acid detergent fibre and acid detergent lignin were determined using sequential analysis, with sodium sulphite, with heat-stable amylase, and expressed inclusive of residual ash. The carbohydrate and protein degradable fractions (non-protein nitrogen; soluble protein; neutral detergent insoluble protein; acid detergent insoluble protein) were estimated according to the Cornell Net Carbohydrates and Protein System CNCPS [36].

### Rumen fatty acid analysis

To determine the FA, each sample (approximately 150 mg) was extracted according to Folch method [37] without drying the final solution containing the lipid extract which was directly methylated using a combination of methods according to Buccioni et al. [2] with the aim to avoid volatile fatty acid (VFA) loss. The first step consisted of an alkaline methylation with sodium methylate/methanol (1 ml of 0.5 M sodium methoxide) to esterify glycerides. The second step involved an acidic methylation using HCl/methanol (1.5 ml of 5% methanolic HCl, 10 min at 50°C) as catalyst to esterify NEFA. Fatty acid methyl esters (FAME) were extracted using n-hexane with C9:0 and C23:0 methyl ester (Sigma Chemical Co., St. Louis, MO) as internal standards for quantification, and maintained in vials with hermetic closure to avoid the loss of volatile components. FAMEs were separated and identified by gas chromatography on a GC equipped with a capillary column (CP-select CB for FAME Varian, Middelburg, The Netherlands: 100 m × 0.25 mm i.d, film thickness 0.20 μm) according to Buccioni et al. [38]. The injector and flame ionisation detector temperatures were 270°C and 300°C, respectively. The programmed temperature was 40°C for 4 min, increased to 120°C at a rate of 10°C/min, maintained at 120°C for 1 min, increased to 180°C at a rate of 5°C/min, maintained at 180°C for 18 min, increased to 200°C at a rate of 2°C/min, maintained at 200°C for 1 min, increased to 230°C at a rate of 2°C/min and maintained at this temperature for 19 min. The split ratio was 1:100, and helium was the carrier gas with a flow rate of 1 ml/min. Individual FAMEs were identified by comparison of the relative retention times of FAME peaks from samples with those from the standard mixture 37 Component FAME Mix C4:0-C24:0 (cod 18919-1AMP, Supelco, Bellefonte, PA, USA), individual trans9 C18:1 and trans11 C18:1 (cod 46903 and v1381, respectively, Sigma-Aldrich, St. Louis, MO, USA), individual cis9, trans11 (cod 1255, Matreya Inc. Pleasant GAP, PA, USA), CLA mix standard (cod 05632, Sigma-Aldrich, St. Louis, MO, USA) and published isomeric profile [39,40]. Determination of the elution sequence of the C18:1 isomers was performed according to Kramer et al. [41]. Moreover, standard mixtures of α-linolenic acid (α-LNA) isomers (47792, Supelco, Chemical Co., St. Louis, MO) and LA isomers (47791, Supelco, Bellefonte, PA, USA) and published isomeric profiles [42] were used to identify the isomers of interest. Two bacterial acid methyl ester mixtures (47080-U Supelco, Chemical Co., Bellefonte, PA, USA; GLC110, Matreya, Pleasant Gap, PA) and individual standards for the methyl esters of *iso* C14:0, *anteiso* C14:0, *iso* C15:0 and *anteiso* C17:0 (21-1211-11, 21-1210-11, 21-1312-11 and 21-1415-11, Larodan Malmo, SW) were used to identify the branched FA profile. Inter- and intra-assay coefficients of variation were calculated using a reference standard (CRM 164, Community Bureau of Reference, Bruxelles, Belgium), and the detection threshold of FA was 0.01 g/100 g of FA [43]. All FA composition results are expressed as g/100 g of FA.

### DNA extraction from rumen microbial samples

Genomic DNA was extracted from 1 ml of rumen microbial suspension using the Fast DNA SPIN kit for soil (Qbiogene, Carlsbad, CA, USA) with some modifications. Briefly, each sample was thawed and transferred to a 15 ml tube containing 4.5 ml of lysis buffer (500 mM-NaCl; 50 mM-Tris–HCl, pH 8.0; 50 mM-EDTA and 4% SDS) and incubated for 15 min at 70°C with gentle shaking by hand every 5 min. After centrifuging at 200 × *g* at 4°C for 5 min, 1 ml of the supernatant was transferred to a 2 ml centrifuge tube and centrifuged at 14,600 × *g* at 4°C for 5 min. The supernatant was removed and the pellet was dissolved in 978 μl of sodium phosphate buffer and 122 μl of MT buffer (both solutions are supplied by the Fast DNA SPIN kit for soil). Each sample was homogenised with a FastPrep cell disrupter instrument (Bio101, ThermoSavant, Qbiogene, Carlsbad, CA, USA) for 2 × 40 s at speed 6.0 and then processed according to the manufacturer’s guidelines. This combination of methods was used to maximise the recovery of DNA from ruminal digests. DNA was eluted in sterile water and its integrity was verified by agarose gel electrophoresis. The amount and purity of DNA was measured at 260 and 280 nm using a ND-1000 Spectrophotometer (NanoDrop Technologies, Labtech, Ringmer, UK).

### PCR-DGGE analysis of the total bacterial community and *Butyrivibrio* group

Individual total DNA extracted from rumen samples was diluted to a concentration of 5 ng/μl and 2 μl of diluted DNA was used as template in PCR reactions. Amplification of the V6-V8 region of the 16S rRNA gene was carried out with the primer pair F968GC (5’-CGC CCG CCG CGC GCG GCG GGC GGG GCG GGG GCA CGG GGG GAA CGC GAA GAA CCT TAC-3’) and R1401 (5’-CGG TGT GTA CAA GAC CC-3’) [44] for total bacterial PCR (fragment size ~470 bp) and with F968GC and Bfib (5’-TTC GGG CAT TYC CRA CT-3’) [45] for *Butyrivibrio* group-specific PCR (fragment size ~470 bp). Reactions were carried out using an iCycler Thermal Cycler (BioRad Laboratories, Hertfordshire, UK) in 25 μl volumes containing 1X PCR buffer (67 mM Tris–HCl, pH 8.8; 1.66 mM (NH_4_)_2_SO_4_; 0.1% Tween-20), 1.5 mM MgCl_2_, 250 μM deoxynucleotide triphosphates (dNTPs), 400 nM each primer, and 1 U of Polytaq (Polymed, Florence, Italy). Amplifications were performed under the following conditions: an initial denaturation at 94°C for 5 min followed by 35 cycles of 94°C for 20 s, 56°C for 30 s and 72°C for 45 s, and a final extension at 72°C for 10 min. After PCR, amplified products were verified by agarose gel electrophoresis. Subsequently, to perform polymerase chain reaction denaturing gradient gel electrophoresis (PCR-DGGE) analysis, amplicons were loaded on a 6% polyacrylamide gel (acrylamide/bis 37.5:1), with a 50-60% denaturing gradient (100% denaturant consisting of 40% v/v deionised formamide, 7 M urea) and electrophoresis was performed in a Phor-U system (Ingeny International, Goes, NL). The gel was run for 17 h at 60°C and 75 V and, after electrophoresis, stained with SYBR® Gold (Molecular Probes, Eugene, OR) and scanned using ChemiDoc XRS (BioRad Laboratories, Hertfordshire, UK).

The PCR-DGGE banding patterns obtained were analysed using the GelCompar II Software v 4.6 software package (Applied Maths, Saint-Martens-Latem, Belgium). Normalisation of bands within and between gels was performed by defining an active reference system. To summarise the species number of rumen bacterial communities, each band was considered as corresponding to a single microbial species. Bands with a minimum area below 1% were discarded. The banding patterns of PCR-DGGEs were further analysed by hierarchical cluster analysis based on the positions and presence/absence of bands in different profiles. The resultant binary matrices were translated into distance matrices using the Dice similarity coefficient and utilised to construct dendrogram using the unweighted pair group method using arithmetic average (UPGMA) algorithm. Analysis of Molecular Variance (AMOVA) was conducted using Arlequin 3.0 software [46], to compare the DGGE patterns and detect statistically significant differences between bacterial community structures in relation to sampling time and diet.

### Sequence analysis of PCR-DGGE fragments

A total of 24 bands were excised from DGGE gels and were placed in 20 μl of distilled water. The PCR products were eluted through freezing and thawing according to Throbäck et al. [18] and reamplified using the F968/R1401 or F968/Bfib primers without a GC clamp, as described above. The fresh PCR products were then sequenced using the dideoxy chain termination method at BMR Genomics sequencing service (BMR Genomics srl, Padova, Italy). Chromatograms were visualised using the Finch TV computer software (ver. 1.4.0, Geospiza, Seattle, WA, USA) and, to obtain reliable results, we carefully verified the absence of many ambiguous peaks in each sequence. Nucleotide sequences were compared against all sequences in GenBank using the BLASTN program [47] with the aim of identifying the microorganisms corresponding to each selected band. Taxonomic identification was achieved by using different sequence similarity thresholds: a similarity ≥97% for a species level identification and 95%, 90%, 85%, 80% and 75% for assignment at the genus, family, order, class and phylum levels, respectively [48].

For phylogenetic analysis, sequences were aligned together with other sequences of equivalent length retrieved from the GenBank database, using the ClustalX 2.0.11 multiple sequence alignment software [49]. Distance calculation was performed according to Jukes and Cantor [50] followed by phylogenetic tree construction using the neighbour-joining algorithm [51] by means of TREECON 1.3b [52]. The robustness of each node was evaluated by bootstrap analysis with 1000 replicates.

### Statistical analysis of fatty acid data

FA concentration data were processed with the General Linear Model of SAS [53] using the following linear model with fixed factors of diet and incubation time as well as their interaction:

$$ {\mathrm{y}}_{\mathrm{i}\mathrm{j}} = \upmu + {\mathrm{D}}_{\mathrm{i}} + {\mathrm{T}}_{\mathrm{j}} + {\mathrm{D}}_{\mathrm{i}} \times {\mathrm{T}}_{\mathrm{j}} + {\mathrm{e}}_{\mathrm{i}\mathrm{j}} $$

where y_ij_ is the observation; μ is the overall mean; D_i_ is the diet (i = 1 to 3); Tj is the incubation time (j = 1 to 3); D_i_ × T_j_ is the interaction between diet and incubation time and e_ij_ is the residual error. Multiple comparisons of means were made using Tukey’s test. Main effects and differences were considered significant when *P* < 0.05.

### Availability of supporting data

Nucleotide sequences from this study have been deposited in the GenBank database. Those from DGGE bands obtained with universal primer pair F968GC/R1401 targeting bacterial 16SrRNA gene have been deposited in the GenBank database under the accession numbers KF976364–KF976382. Those from DGGE bands obtained with primer pair F968GC/Bfib specific for the *Butyrivibrio* group have been deposited in GenBank under accession numbers KF976383-KF976387.

Band matching tables of Bacteria and *Butyrivibrio* DGGE profiles according to diet and time of sampling have been deposited to LabArchives, LLC (http://www.labarchives.com/) at https://dx.doi.org/10.6070/H4HH6H16.

## Abbreviations

α-LNA: α-linolenic acid
BH: Biohydrogenation
CALNA: Conjugated linolenic acid
CLA: Conjugated linoleic acids
FA: Fatty acids
FAME: Fatty acid methylesters
LA: Linoleic acid
OA: Oleic acid
PCR-DGGE: Polymerase chain reaction denaturing gradient gel electrophoresis
RA: Rumenic acid
RL: Rumen liquor
SA: Stearic acid
SOP: Stoned olive pomace
VA: Vaccenic acid
VFA: Volatile fatty acids
VLA: Vaccelenic acid
